# Experimental investigation of four-port MIMO-Dual-Band (MDB) antenna for n260/n263 mmWave bands with analysis including conformal & specific-absorption-rate

**DOI:** 10.1371/journal.pone.0350033

**Published:** 2026-05-29

**Authors:** Manish Sharma, Tathababu Addepalli, Gaurav Sharma, Tanweer Ali

**Affiliations:** 1 Department of Electrical, Electronics and Communication Engineering, Galgotias University, Greater Noida, Uttar Pradesh, India; 2 Department of Electronics and Communication Engineering, Aditya University, Surampalem, Andhra Pradesh, India; 3 Department of Electronics and communication Engineering, Saveetha School of Engineering, Saveetha Institute of Medical and Technical Sciences, Thandalam, Chennai, Tamilnadu, India; 4 Centre for Research Impact & Outcome, Chitkara University Institute of Engineering and Technology, Chitkara University, Rajpura, Punjab, India; 5 Manipal Institute of Technology, Manipal Academy of Higher Education, Manipal, India; Model Institute of Engineering and Technology, INDIA

## Abstract

The single-input-single-output (SISO) system or the single-port antenna faces the problem of path-loss and fading of the signals, which results in loss of data. However, the fast-data transmission is needed in an hour, and the multiple-input-multiple-output millimeter-Wave (MIMO_mmWave_) technology satisfies the high-speed of communication without a low loss of data, irrespective of the design of the antenna. The millimeter-Wave bands need to be explored since the lower 5 G-sub 6 GHz is already overloaded with huge traffic and is unable to cater to the needs. Hence, to utilize millimeter-Wave bands for n260 and partial-n263 in FR2, a four-port MIMO_mmWave_ antenna is designed. The proposed MIMO_mmWave_ work is characterized for S-parameters, far-field results, bending analysis, and SAR calculation for on-body applications. This research article discusses ultra-compact dual-band MIMO_mmWave_ antenna radiating EM Energy is designed for n260 and partial-n263 band with etched circular-patch printed on top-surface of 0.254 mm thickness Rogers-flexible substrate and ground on opposite surface. The single-port version occupies PCB space of 6 × 8 mm2 (0.93λ_0_×1.23λ_0_), and the four-port proposed version utilizes 12 × 16 mm^2^ (1.85λ_0_×2.47λ_0_) space. The fabricated four-port mmWave antenna achieves impedance bandwidth of 36.60GHz-39.12GHz and 58.48GHz-61.48GHz. The MIMO antenna also offers an averaged gain in n260 and partial-n263 band corresponding to 6.532dBi & 6.978dBi. The conformal capability at 45° in the x-axis and y-axis maintains both the mmWave bands without much deviation in comparison with planar no-bending of the MIMO_mmWave_ antenna. The diversity parameter for n260/ partial-n263 bands corresponds to Envelope Correlation Coefficient<2.78 × 10^-4^ (38.0GHz)/2.93 × 10^-4^ (60.0GHz), Diversity Gain_mmWave_≈10.0dB (38.0GHz/60.0GHz), Total Active Reflection CoefficientmmWave < −7.358dB (38.0GHz)/-9.12dB (60.0GHz), Channel Capacity LossmmWave<1.58 × 10^-2^ (38.0GHz)/1.83 × 10^-2^ (60.0GHz) and Mean Effective Gain_mmWave_ (all ports)≈-30.0dB (38.0GHz/60.0GHz) and is deployable in 5G infrastructure. The SAR analysis with respect to single port corresponds to 0.0174W/Kg at 38.0GHz and 0.0384W/Kg at 60.0W/Kg and the MIMOmmWave values are 0.00895W/Kg at 38.0GHz & 0.0301W/Kg at 60.0GHz.

## 1. Introduction

In the current scenario, wireless communication plays a vital role in the modern era. The complete communication module deployed has emerged with the best solution to transfer data rate, which can be either voice, video, or both. To achieve this concept of a higher data rate of transmission, a higher bandwidth is essential. The objective of the work is, hence, to produce a MIMO-Antenna with millimeter wave characteristics, and the supporting literature with respect to 38.0GHz & 68.0GHz is discussed. The literature is focused on dual-band MIMO technology, with [1,2] focused on a single resonance centered at 38.0GHz band. A 38.0GHz is achieved by etching a rectangular slot on top of a circular patch [1] with partial ground. The identical patches are then placed in orthogonal sequence, transforming a single-port to a four-port configuration with an overall size of 25.95 × 28.95 mm^2^. The array-concept is used [2] in 37.0GHz MIMO configuration to achieve gain of 6.84dBi and is raised to 12.80dBi by using array-concept. Dual-band with mmWave frequencies, including 28.0GHz and 38.0GHz, are discussed [3–10] with a crescent-shape radiator modified with an inset-feed & etched slots. This achieves resonances at 28.0GHz and 38.0GHz. The better diversity performance is achieved by placing the four-radiators orthogonally [3]. The four-port MIMO antenna with the patch initiated from a rectangular structure uses complimentary-split-ring-resonator structure, which helps in improving gain and directivity [4]. One way of achieving dual-band is to generate resonances at desired frequencies, and the other method is to achieve a wide bandwidth. A F-type structure forming a radiating-patch and partial-ground with identical F-structure offers a wide impedance-bandwidth with integrated 28GHz/38GHz bands [5]. A perforated-patch on top of the substrate and defected-ground achieves 0.60GHz bandwidth with f_res_ = 28.0GHz and 1.17GHz bandwidth with corresponding f_res_ = 38.0GHz [6]. Pair of T-type radiators with one corresponding to resonance at 28.0GHz and the other corresponding to 38.0GHz [7–10] provides highly directive radiation-patterns in boresight directions for 5G mobile phone applications. As discussed earlier, the objective of the work is to achieve a dual-resonance bandwidth of 38.0GHz [1–10] and 60.0GHz bandwidth [11–27]. The resonance at 60.0GHz is achieved by using an Artificial-Magnetic-Conductor to achieve high gain, and a dipole-structure achieves a bandwidth of 57.0GHz-64.0GHz [11]. Stepped-Impedance-Resonator shows the capability of generating V-band and W-band with broadside radiation gain of 7.90dBi and 4.20dBi, respectively [12]. The array-configuration of 5G-mmWave with rectangular-patch [13–17,28,29] is reported at 60.0GHz with a maximum gain of 15.52dBi and offers highly directive 2-D radiation patterns. A combination of rectangular-patch with etched half-circular-slot and ground etched with circular-slot resonates at 60.0GHz, and the array-configuration generates a gain of around 15.0dBi [18–19]. The 60.0GHz resonance is also achieved by etching a Q-slot on an asymmetrically-fed rectangular-patch and ground [23]. A propagation-model at 60.0GHz is reported [25] with free-path loss increases as the frequency increases. The penetration loss is also reported at 60.0 GHz, with a penetration loss of 10.20 dB. A flexible-antenna designed on Rogers RTDuroid 5880 substrate provides bandwidth between 7.20GHz-9.20GHz, and the performance is evaluated for on-body applications [30]. A super-wideband antenna with bandwidth 1.66GHz-160.0GHz is the result due to an etched circular-slot and an etched partial-rectangular-slit [26]. The DRA of height 285 mm is placed above the patch with a maximum gain reaching 13.20dBi. A review on 5G-communication is reported [31] with a discussion of budget-link and the integration in semiconductor technologies. A review on conformal antenna [32,33] is discussed with various substrates, such as glass-epoxy, transparent-substrate, FR4, and Rogers substrate, which are used with a thickness≤0.254mm. A dual-band MIMO antenna offering two-narrow bands with resonances coinciding at 38.0GHz and 60.0GHz is achieved by using two electromagnetically coupled patches [34–38].

The objective of the paper is to design dual millimeter-Wave single and four-port antennas in millimeter-Wave FR2 bands. The patch comprises of circular-etched patch with ground printed on opposite planes. This configuration of the antenna results in n260 (38.0GHz) and partial-n263 (60.0GHz) mmWave bands designed on the thin substrate. The radiating patches are placed adjacent to each other with 180° rotation, achieving spatial diversity. The antenna is also subjected to conformal analysis with bending in the x-axis and y-axis. The SAR_mmWave_ is also calculated for 38.0GHz and 60.0GHz by using a tissue model. The detailed analysis is discussed below, with Section 2 discussing the details of the single-port antenna, Section 3 focusing on Conformal and SAR analysis of the single-port, Section 4 giving details on the four-port mmWave MIMO antenna, and Section 5 giving insight into Conformal, SAR, and Far-Field Analysis of MIMO_mmWave_ antenna.

The proposed work contributes more than the not just the size reduction nut also includes the distinct contributions listed below.

(i) The realization of the dual FR2 bands: n260 and n263
(ii) Four-port common-ground MIMO architecture on ultra-thin (0.254 mm) Rogers flexible substrate without employing split-ground or complex decoupling structures.
(iii) Full bending analysis in both orthogonal x-axis and y-axis with bending angle upto 45°
(iv) The study of SAR simultaneously for both single and four-port MIMO antennas at 38.0 GHz and 60.0 GHz

## 2. 1-port antenna

**Fig 1:**
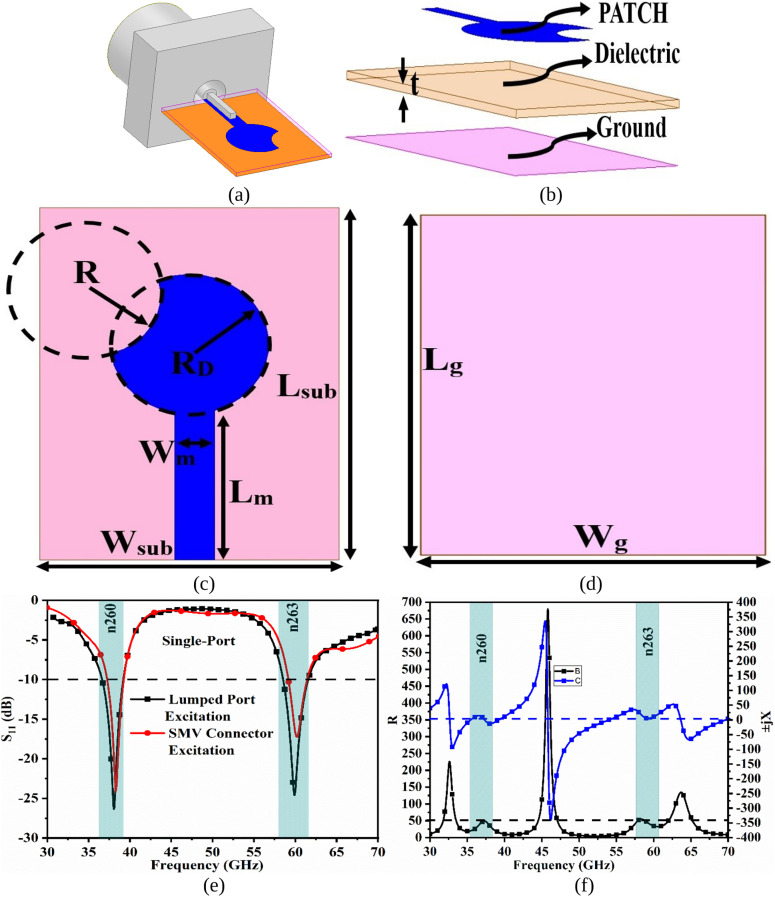
1-Port mmWave Configuration. (a) 3-D view (b) Patch details (c) Ground details (d) S_11_ plot (e) Impedance-graph.

The objective of the work is to design a compact MIMO-Dual-Band (MDB) which can resonate at 38.0GHz (n260) and 60.0GHz (n263) bands. Fig 1 gives the insight of the design with Fig 1(a), 1(b) shows the 3-D model of the proposed antenna, where Fig 1(a) corresponds to a simulator modeled connector connected to the patch antenna to understand the influence of the connector and the transition modeling. The antenna consists of three components patch, a dielectric, and a ground. For the signal transmission, the SMK connector was modeled in a simulator with a matching impedance corresponding to 67.0GHz. The very thin dielectric material with thickness t = 0.254 mm is used in the design, which can be utilized for conformal applications corresponding to material Rogers RTDuroid5880 with permittivity = 2.20. Fig 1(c) and 1(d) show the radiating patch printed on the top plane of the dielectric and ground on the opposite side. The circular-patch is etched partially by the external circle of radius R mm as observed in Fig 1(c) to achieve dual narrow resonance. The ground dimensions, L_g_ × W_g_ mm^2,^ which are printed on the opposite side of the patch is equal to the dimensions of the substrate L_sub_ × W_sub_ mm^2^ to achieve narrow-bandwidth. Further, the simulation results are plotted in Fig 1(e) and 1(f), with Fig 1(e) corresponding to the S_11_ plot, and the antenna input port is swept between 30.0 GHz and 70.0 GHz, with the simulations including the S_11_ achieved by Lumped-port excitation and the SMV connector excitation. The first narrow-band corresponds to n260 with a bandwidth of 35.95GHz-38.44GHz and offers a total impedance of (35.83-j12.63) Ω at 38.0GHz. Similarly, the second narrow-band, partial-n263, corresponds to a bandwidth of 57.87GHz-60.79GHz with a total impedance of (34.57 + j10.34) Ω at 60.0GHz. The two impedance values at 38.0GHz and 60.0GHz suggest the capacitive and inductive nature of the proposed work, but very little energy is stored. Also, the connector modeled S_11_ results correspond to two dual bands with bandwidths of 37.23 GHz-39.31 GHz (resonance = 38.01 GHz; S_11_ = −24.20 dB) and 29.07 GHz-61.41 GHz (resonance = 60.11 GHz; S_11_ = −17.25 dB). The optimal dimensions are obtained from the EM-simulator and are given below in Table 1

**Table 1:** Optimal Dimensions (Corresponds to Fig 1).

W_sub_	6.00	R_D_	1.80
L_sub_	8.00	R	1.36
W_m_	0.80	L_g_	8.00
L_m_	3.50	W_g_	6.00

Furthermore, to investigate the single-port mmWave antenna, the surface-current-density (SCD_mmWave_), parametric study of key-parameters, 2-D radiation patterns, and gain-frequency plot are studied. This investigation will help in achieving the objective of the four-port mmWave dual-band antenna.

**Fig 2:**
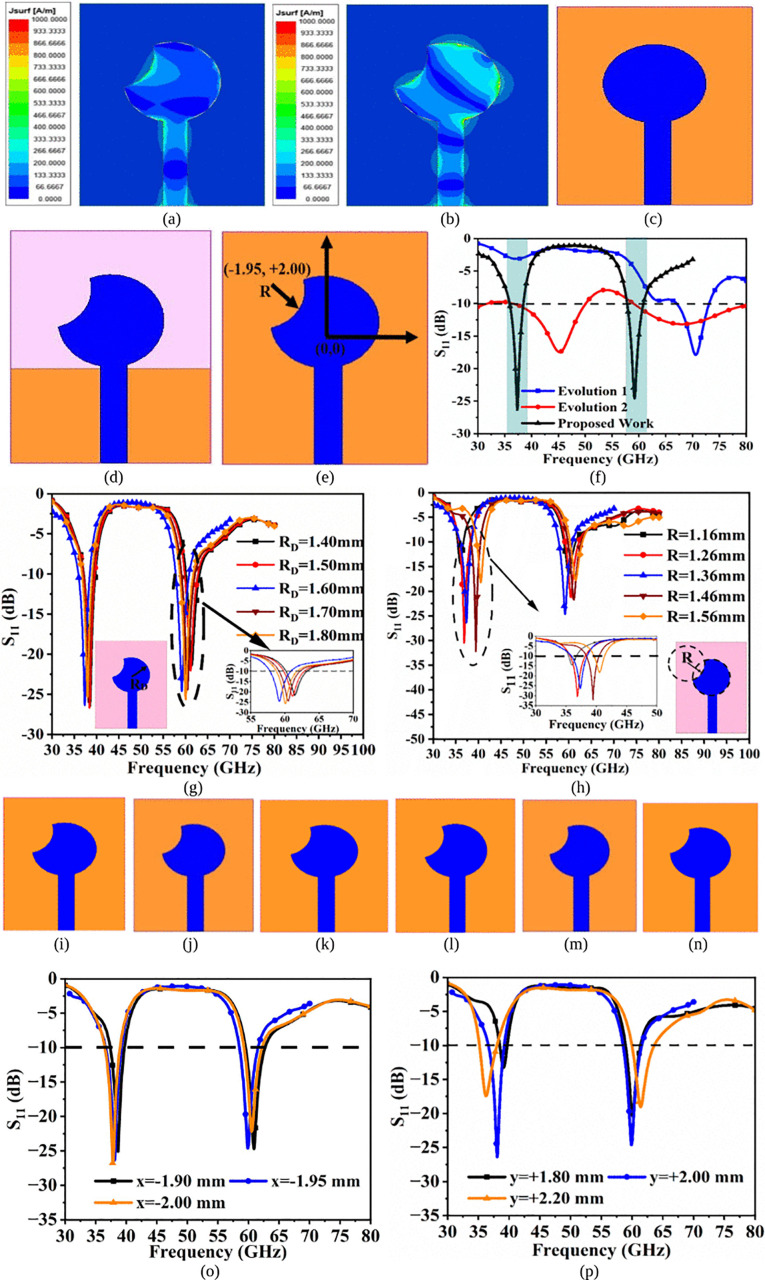
SFD_38.0/60.0GHz_ at (a) 38.0GHz (b) 60.0GHz (c) Evolution 1 (d) Evolution 2 (e) Proposed Work (f) S_11_-Evolution Comparison; Parametric study (g) R_D_ (h) R; co-ordinate value (i) x = −1.90 mm (j) x = −1.95 mm (k) x = −2.00 mm (l) y=+1.80 mm (m) y=+2.00 mm (n) y=+2.20 mm (o) S_11_ values with respect to variation of x-axis (p) S_11_ values with respect to variation of y-axis‌‌.

Fig 2 gives an illustration and more detailed insight into the development and evolution of the single-port dual-band mmWave antenna. Fig 2(a) and 2(b) shows the surface-current-density distribution at 38.0GHz (n260) and 60.0GHz (n263) bands. The SFD_38.0/60.0GHz_ is carried out on a scale between 0 and 1000 A/m. The SFD_MDB_ at 38.0GHz shows that the antenna radiates maximum signal due to the good-impedance match of (35.83-j12.63) Ω. Also, the SFD_DB_ at 60.0GHz with impedance of (34.57 + j10.34) Ω also confirms the radiation of the antenna when signals are fed to it. Significantly, the evolution of the proposed work is represented in Fig 2(c), 2(d), 2(e) and the corresponding S_11_ parameter are plotted in Fig 2(f). The approximate resonance frequency is calculated from Equation 1, Equation 2, and Equation 3.

Fig 2(c) shows the initial-port antenna with a circular patch and substrate-sized ground, and the radius is calculated by [39]


$$\begin{array}{c} f_{r}=\frac{\text{1}.\text{8}\text{4}\text{1}\text{2}\times c}{\text{2}\times \pi d_{eff.}\sqrt{\varepsilon_{eff.}}} \end{array}$$ (1)

$$\begin{array}{c} f_{r}\propto \frac{\text{1}}{d_{eff.}} \end{array}$$ (2)

$$\begin{array}{c} f_{r}\propto \frac{\text{1}}{\text{R}} \end{array}$$ (3)

where c = 3 × 10^8^ m/s, a_eff._ and ε_eff._ Corresponds to the effective radius of the circular-patch and the permittivity of the dielectric material.


$$\begin{array}{c} \frac{R_{D}}{\text{2}}=\frac{d}{\sqrt{\left(\text{1}+\frac{\text{2}t}{d\pi \varepsilon_{r}}\left[ln\left\{\frac{\pi d}{\text{2}t}\right\}+\text{1}.\text{7}\text{7}\text{2}\text{6}\right]\right)}} \end{array}$$ (4)

$$\begin{array}{c} d=\frac{\text{8}.\text{7}\text{9}\text{1}\times {\text{1}\text{0}}^{\text{9}}}{f_{res.}\sqrt{\varepsilon_{r}}} \end{array}$$ (5)

$$\begin{array}{c} d_{eff.}=\frac{R_{D}}{\text{2}}\;\sqrt{\text{1}+\frac{\text{2}t}{\pi R_{p}\left[ln\frac{\left(R_{D}/\text{2}\right)}{\text{2}t}+\text{1}.\text{7}\text{7}\text{2}\text{6}\right]}} \end{array}$$ (6)

Equation 4, Equation 5, and Equation 6 calculate the physical radius and effective radius of the circular-patch antenna. This antenna provides a resonance bandwidth of 67.0GHz-73.11GHz with f_resonance_ = 70.64GHz with S_11_ = −17.88dB. The second evolution shows the etching of the circular-patch and the partial-ground-plane. This evolution corresponds to two dual-bands, 36.45GHz-50.10GHz and 59.07GHz-80.0GHz. The two resonance values at these two-bands corresponds to 45.16GHz with S11 = −17.46dB and 68.04GHz with S_11_ = −17.17dB. The third iteration utilizes a full ground with the same patch used in the previous iteration. This is the proposed work with dual-bands, 35.92GHz-38.48GHz with S_11_ = −26.36dB and 57.80GHz-60.88GHz with S_11_ = −24.60dB. The Equation (1) corresponds to the fundamental TM_11_ mode resonance where the circular-patch is the cavity-model generating the 38.0 GHz resonance. Also, both resonances, 38.0 GHz and 60.0 GHz, are generated simultaneously, 38.0 GHz by the circular-patch of radius RD = 1.80 mm and the 60.0 GHz by an etched partial inner-circular patch of radius R = 1.36 mm with full ground responsible for generating dual narrow millimeter-wave bands. Table 2 illustrates the summary which relates the dimensions and the two-narrow bands where the key parameter designs, RD, R, size of ground, and thickness of substrate (t), controlling the resonances, 38.0 GHz & 60.0 GHz.

**Table 2:** Relation between dimensions and dual resonance(s).

Related-parameter	Effect	Frequency control
RD (radius of patch)	The transverse-magnetic (TM_11_) is controlled	Corresponds to 38.0 GHz
Inner etched circular-patch of radius R	Perturbed higher-mode or slotted-mode is controlled	Corresponds to 38.0 GHz
Dimension of the ground	Bandwidth and impedance matching are affected	Dual narrow-bands
Height of the substrate (t)	Bandwidth as well as Q-factor are affected	Dual narrow-bands

Furthermore, to understand the effect of a circular patch of radius R_D_ mm and an etched circular slot of radius R mm is studied in Fig 2(g) and 2(h). The radius, R_D_ mm, of the etched radiating-patch has an impact on the second resonance at 60.0GHz. when the value of R_D_ is varied between 1.40 mm and 1.80 mm with a step size of 0.10 mm. It can be seen that from Fig 2(g), the resonance shifts from higher to lower side, and for R_D_ = 1.80 mm, the antenna resonates at 60.0GHz. The second-parameter, R mm, which is used to etch the circular-patch effects the first band (n260), and the change in Radius R mm from 1.16 mm to 1.56 mm also shifts the resonance from higher to lower. The value of R = 1.36 mm corresponds to a resonance frequency of bandwidth of 35.92GHz-38.48GHz. The n260 band is widely used for 5G-NR networks for short-range communication, such as applications in scaling of larger events for more than thousands of users, vehicle-communication (Transport System), Monitoring of Environment, Smart Cities, and improved residential connections are a few to name them. On the other hand, the partial-n263 band finds its applications in smaller cells for indoor applications, as the attenuation increases at very high frequencies.

Fig 2 also includes how sensitive the resonant frequencies are to slot width; feed position affects the resonance at 38.0 GHz and 60.0 GHz, respectively.


$$\begin{array}{c} \lambda_{g}=\frac{c}{f_{\text{6}\text{0}.\text{0}\;GHz}\sqrt{\varepsilon_{eff.}}}\approx \text{3}.\text{0}\;mm \end{array}$$ (7)

Equation 7 calculates the guide-wavelength at 60.0 GHz which is 3.0 mm and, hence the variation of ±0.05 mm records a change of approximately 1.7% which is merely non-negligible which is related to sensitivity. Fig 2(i), 2(j), 2(k), 2(l), 2(m), 2(n) show the change in position of the inner etched circular patch of radius R with respect to the x-axis and y-axis. Considering the center of the designed antenna at (0,0) as shown in Fig 2(e), the inner slot is moved along the x-axis for three positions of x-axis, x = −1.90 mm, 1.95 mm, and 2.00 mm, with respective S_11_ results recorded in Fig 2(o). The resonance frequency at 38.0 GHz and 60.0 GHz is shifted from their desired resonance for values of x = −1.90 mm and −2.00 mm, whereas the value of x = −1.95 mm maintains the two resonance values, which is the fixed position of x. Similarly, Fig 2(p) shows that the resonance frequency at 38.0 GHz and 60.0 GHz are shifted from their desired resonance for values of y=+1.80 mm and +2.20 mm, the value of y=+2.00 mm maintains the two resonance values, which is the fixed position of y.

## 3. 1-port conformal and SAR analysis

**Fig 3:**
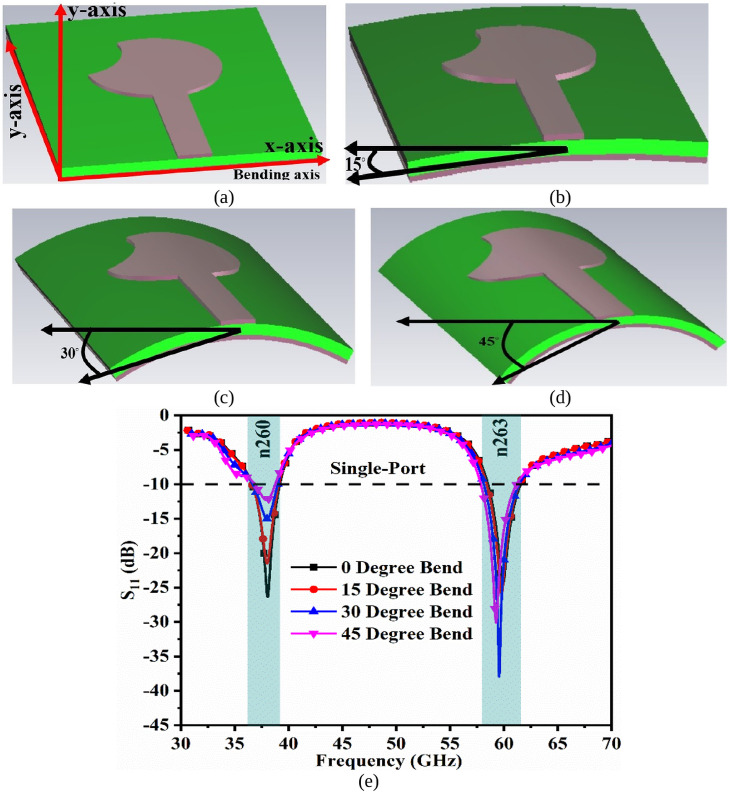
Bending of the antenna with respect to the x-axis. (a) 0° or no-bending (b) 15° (c) 30° (d) 45° (e) S_11_ plot for all angled-bending.

The bending of the proposed work is shown in Fig 3 with bending analysis carried out at 0° (no-bending), 15°, 30° and 45°. This bending of the antenna is also known as conformal analysis, with the bending of the antenna being subjected to both edges in the x-axis direction, as shown in Fig 3(a). The planar-antenna shown in Fig 3(a) (no-bending) offers impedance dual-narrow bandwidth corresponding to 36.64GHz-39.02GHz (S_11_ = −25.98dB at 38.0GHz) and 58.52GHz-61.56GHz (S_11_ = −24.60dB at 59.88GHz as noted from Fig 3(e).

Fig 3(b), 3(c), 3(d) show the bending of the antenna at 15°, 30° ad 45° with a maximum bending of 45° is enough for conformal applications. At 15° degree bending, the dual bands correspond to 36.65GHz-39.12GHz (S_11_ = −21.51dB at 38.0GHz) and 58.40GHz-61.48GHz (S_11_ = −25.87dB at 59.80GHz). The antenna with a bending angle of 30° achieves the first band of 36.68GHz-39.40GHz (S_11_ = −15.01dB at 38.0GHz) and 58.12GHz-61.44GHz (S_11_ = −37.95dB at 59.56GHz). The final-bending is applied at 45° achieving a first-narrow bandwidth of 36.88GHz-38.80GHz and 57.84GHz-61.16GHz with maximum resonance at 37.96GHz (S_11_ = −12.29dB) and 59.28GHz (S_11_ = −30.12dB). The conclusions from Fig 3(e) can be summarized as follows.

(a) At the n260 band, the resonance value with respect to S_11_ changes for all four angles of bending, with impedance-matching becoming poor but well below −10.0dB. All the four-narrow-bands achieved in bending are within the specified n260 bandwidth of 37.0GHz-40.0GHz.
(b) For the second-narrow of n263, the observations are that the resonance frequency points do deviate as observed in Fig 3(e), but the bandwidth in all four cases is within the n263: 57.0GHz-71.0GHz bandwidth.

**Fig 4:**
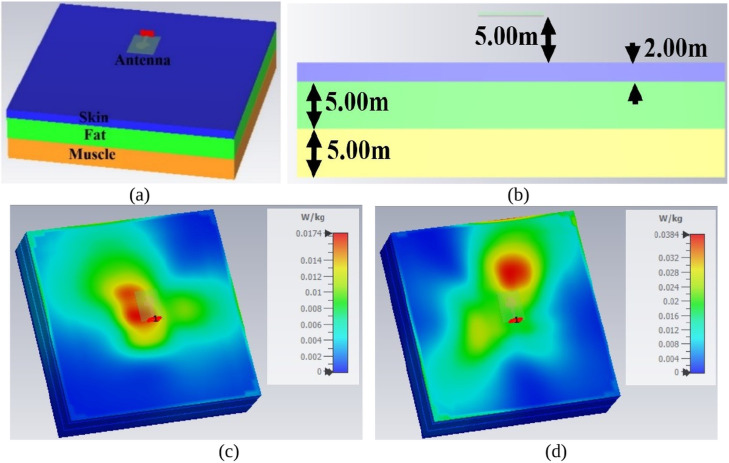
Different views of the tissue-model. (a) Isometric view of tissue model (b) Side view with antenna; Specific-absorption-rate analysis at (c) 38.0GHz (n260) (d) 60.0GHz (n263)‌‌.


At the millimeter-wave resonance frequencies, the penetration of the electromagnetic-signals is shallower (deep penetration), which can be of the order of 0.30 mm to 0.80 mm. However, the ICNIRP compliance states that the values for general public exposure must not exceed 10 W/m^2^. However, the SAR values calculated are well below the prescribed compliance values. Fig 4(a), 4(b) shows the tissue-model of the antenna with isometric and side views. The isometric view shows the three layers of the tissue model subjected to analysis of SAR with an antenna placed above the model. The thickness of the skin, fat, and muscle corresponds to 2.00 mm, 5.00 mm, and 5.00 mm. The selection of the material, such as FR4, Rogers RTDuroid, Polytetrafluoroethylene (PTFE), Poluethylene-Terethalate (PTE) are few dielectric substrates that are used for on-body applications. In the above discussion, the conformal analysis was discussed with bending at 0°, 15°, 30° and 45°. In all four cases, the bandwidth requirement is not compromise which means that the single-port antenna can easily be integrated with devices for on-body applications. The key in designing the conformal antenna for on-body applications is the thickness of the substrate used. The above-mentioned dielectric materials are available with t≤0.254mm, which can be easily converted to conformal. Fig 4 shows the analysis of the single-port antenna, which is placed on the human-tissue and is simulated for the 38.0GHz/60.0GHz SAR_38.0/60.0GHz_ analysis. The specific-absorption-rate (SAR_38.0/60.0GHz_) is frequency dependent parameter and depends on the tissue conductivity (S/m), E the electric field intensity, loss tangent of each layer of the tissue, and the tissue mass (kg/m^3^). The Equation 8 used in the calculation of the SAR_38.0/60.0GHz_ equation is given below.

$$\begin{array}{c} {SAR}_{\text{3}\text{8}.\text{0}/\text{6}\text{0}.\text{0}GHz}=\int \frac{\sigma (r){\left|E(r)\right|}^{\text{2}}}{\rho (r)}dr \end{array}$$ (8)

The values of conductivity, loss tangent, and permittivity do change with the change of frequency and are tabulated in Table 3, given below

**Table 3:** Electrical properties of the tissue.

Tissue/Frequency	38.0GHz			60.0GHz		
	Permittivity (ε_r_)	Conductivity (S/m)	Loss Tangent (tan δ)	Permittivity (ε_r_)	Conductivity (S/m)	Loss Tangent (tan δ)
**Skin**	123	31.0	1.7941	7.98	36.4	1.3673
**Fat**	5.33	6.36	0.29331	4.40	8.39	0.26925
**Muscle**	19.1	41.8	1.0382	12.9	52.8	1.231

The electrical parameter such as permittivity, conductivity, and loss tangent is derived from [40,41]. The mass density for skin, fat, and muscle corresponds to 1109 kg/m^3^, 911 Kg/m^3,^ and 1090 kg/m^3^. The condition applied in the calculation of SAR_38.0/60.0GHz_ is with the input power of 50mW, and 1g of the tissue model is considered in the simulation. As per the observations from Fig 4(c) and 4(d), the SAR_38.0/60.0GHz_ values correspond to 0.0174 W/kg for 38.0GHz and 0.0384 W/kg for 60.0GHz. These two values are less than the standard values of 1.60W/kg, which suggests that the single-port antenna is suitable for on-body applications at mmWave frequencies.

## 4. 4-port mmWave MIMO_38.0/60.0GHz_ configuration

**Fig 5:**
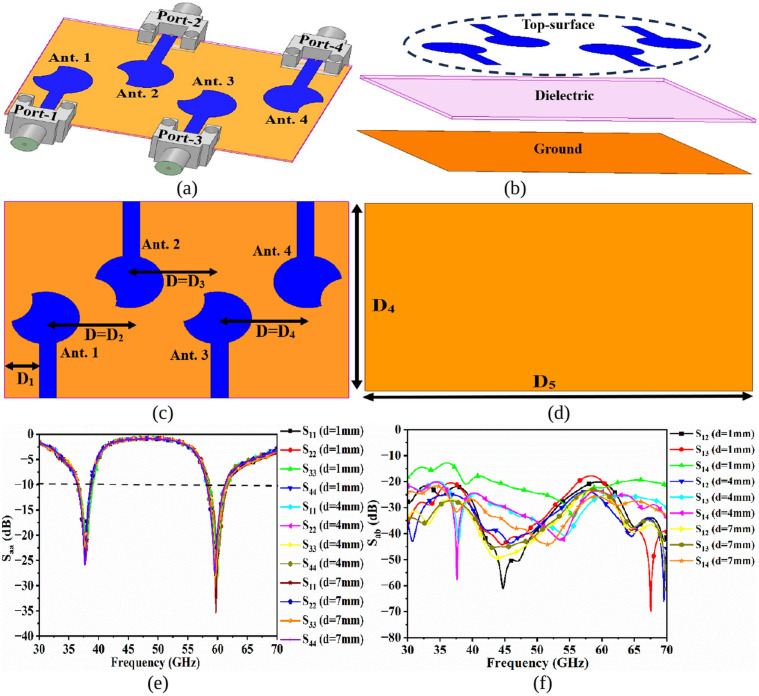
4-Port MIMO_38.0/60.0GHz_ Antenna Configuration (a) Isometric model (b) Structure; Dimensions (c) FV (d) GV.


Fig 5 shows the 4-Port MIMO_38.0/60.0GHz_ antenna configuration for high-speed wireless communication data transfer. Fig 5(a) gives the details of the antenna with four-radiating elements sharing a common ground. The proposed work overcomes the problem of non-common ground for each radiating element, as the size of the ground is the same as the size of the substrate [42]. In many of the cases, the isolation is achieved with a separate ground, which achieves isolation, and this is due to the fact that there is no coupling path through the ground. The split in the ground is not practical, and hence, a common ground is always required so that the signal levels can be analyzed practically. Fig 5(b) shows the three components of the proposed four-port MIMO_38.0/60.0GHz_ with radiating patch comprising four patches, substrate, and ground, which form the antenna system. Fig 5(c) shows the front-view of the antenna with detailed interspacing between the elements. The distance D_1_ = 1.50 mm is the dimension between the edge of the substrate and the microstrip feed line of Ant. 1, and the interspacing between the radiating elements is D = D_2_ = D_3_ = D_4_ = 4.00 mm. The total dimension of the antenna is the same as the size of the substrate, which is also the size of the ground, as shown in Fig 5(d), occupies the area of D_4_ × D_5_ = 12.0 × 16.0 mm^2^. Fig 5(e) and 5(f) record the S-parameters when the interspacing distance D is changed with values of D = 1.0 mm, 4.0 mm, and 7.0 mm. The values of D = 1.0 mm and 7.0 mm record the deterioration in the isolation, whereas the impedance bandwidth generated by each radiating element for both the values ensures the 38.0 GHz and 60.0 GHz millimeter wave bands. In a Single-Input-Single-Output (SISO) system, the working or deployment of an antenna in a multipath fading environment suffers fading of the signal at the receiver side, where the receiver receives signal from different impinged angles and thereby generates constructive or destructive signals. In the latter case, the signal is lost, and hence deteriorates the continuous communication and results in a loss of information. To overcome the above scenario, printing a greater number of radiating elements at both the transmitter and receiver will solve the problem of fading. Thus, the multiple-input-multiple-output technology is used to combat the issues of fading of the signals by transmitting the data from multiple antennas and receiving the same by an identical MIMO antenna. In a MIMO system, the transmitted signal travels in different paths and thus the probability of receiving the signal in the fading-environment increases. The capacity of the channel is also increased when the number of radiators using diversity schemes increases, which is given by Equation 9

$$\begin{array}{c} C_{Ch.\;Cap.}=M_{no.\;of\;elements}B_{BW}{log}_{\text{2}}\left(\text{1}+\frac{N_{RX}}{M_{TX}}SNR\right) \end{array}$$ (9)

Where C_Ch. Cap_ is the capacity of the channel in bits/sec, M_TX_ and N_RX_ correspond to the number of radiating elements in the transmitting and receiver antenna, BBW is the operational bandwidth, and SNR signifies signal-to-noise ratio, which should be large enough [43].

**Fig 6:**
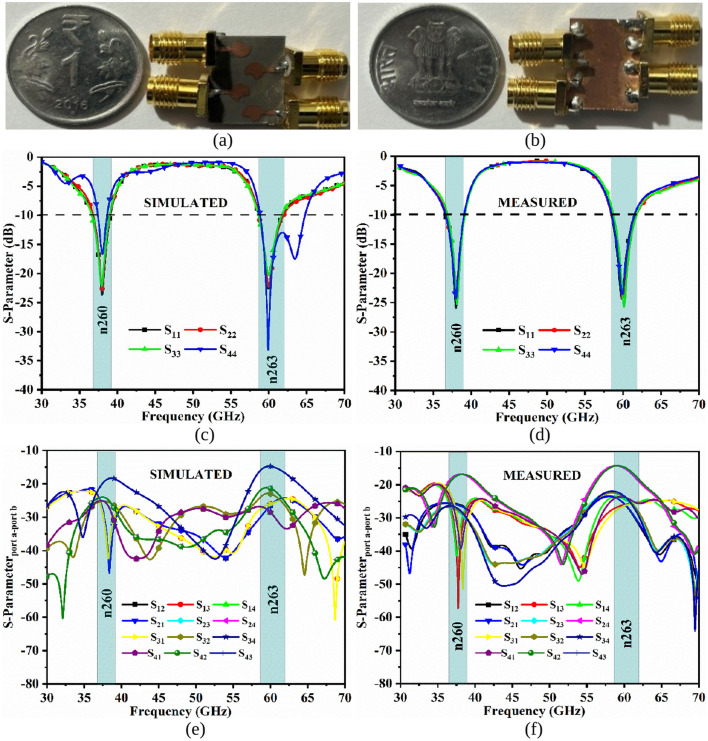
4-Port MIMO_38.0/60.0GHz_ Antenna Configuration (a)-(b) Fabricated radiating-ground view; Simulated and Measured (c)-(d) Reflection Coefficient (e)-(f) Transmission coefficient.

Fig 6 shows the photo of the prototype with simulated and measured S-parameters for all four ports (S_11_/S_22_/S_33_/S_44_). Fig 6(a) shows the front-view of the MIMO antenna with the arrangement of radiating patches in such a sequence that the radiation-patterns interference is very little. The ground-view is shown in Fig 6(b), which signifies the size of the ground being equal to the size of the substrate for extracting narrow-bandwidth. Fig 6(c) and 6(d) record the value of S-parameter for all the four-ports in the simulation and measurement scenario. In case of S-parameter for Ant. 4 (Fig 5), the bandwidth is widened for the partial-n263 band, whereas for the remaining radiating patches (Ant. 1, Ant. 2, and Ant. 3), the required bandwidth of 37.00GHz-40.0GHz and 57.0GHz-71.0GHz is achieved. In the case of measured S-parameters, all four patch results in the required dual-narrow bands. Fig 6(e) and 6(f) records the isolation between any two-port (S_12_, S_13_, S_14_, S_21_, S_23_, S_24_, S_31_, S_32_, S_34_, S_41_, S_42_ and S_43_). The simulation and measured S-parameters show that the inter-port isolation is more than 15.0dB.

In a multiple radiating antenna system, the diversity schemes are utilized, which can be spatial diversity, pattern diversity, or polarization diversity. The spatial diversity scheme is used in the proposed work, where the radiating elements are spaced such that minimal interference is experienced by each of the radiating elements.

Isolation (S_ab_, S_ba_) corresponds to the measurement of power, which is coupled with the neighbouring radiating elements. The isolation is improved by using techniques such as orthogonal orientation of the radiating elements or by placing the radiating antenna at least spacing more than λ/2 between them or by using other isolation techniques such as addiing stub, etching slots, using meta-material, or using electromagnetic-band-gap structure.

The experimental setup was conducted, where the measurement procedure, post-processing method of calculating diversity parameters, and the uncertainty connected to measurement are discussed. The measurements of reflection-transmission coefficients were extracted by connecting the ports of the proposed MIMO antenna using a Vector Network Analyzer (VNA) with measurement capability between 30.0 GHz-70.0 GHz and was calibrated before measurement by following the Short-Open-Load-Through, also known as SOLT calibration process. The cables, due to the designed MIMO antenna ranging in millimeter wave frequency bandwidth, low-loss high frequency rated up to 67.0 GHz, are used. The parasitic reflections are also taken care by terminating the two port with perfect terminating 50Ω load whereas the remaining two-port are connceted by high quality connector with minimal losses. This setup of the MIMO antenna connected to the VNA facilitated the measurement of S-parameters with more accuracy. The extracted S-parameters were used to obtain the diversity parameters, including ECC_mmWave_, DG_mmWave_, TARC_mmWave_, CCL_mmWave,_ and MEG_mmWave_.

**Fig 7:**
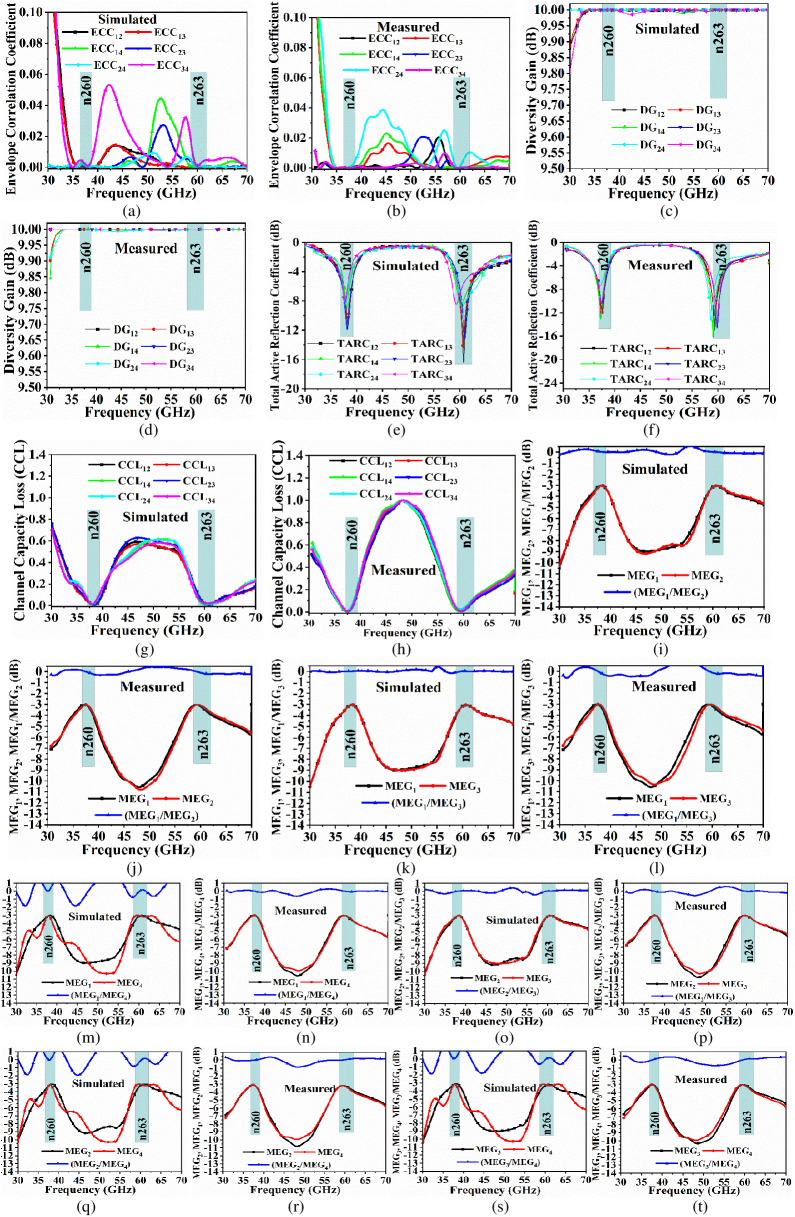
4-Port MIMO_38.0/60.0GHz_ Antenna Diversity Parameters. (a)-(b) Simulated and Measured ECC_38.0/60.0GHz_ (c)-(d) Simulated and Measured DG_38.0/60.0GHz_ (e)-(f) Simulated and Measured TARC_38.0/60.0GHz_ (g)-(h) Simulated and Measured CCL_38.0/60.0GHz_ (i)-(t) Simulated and Measured MEG_38.0/60.0GHz_ (port1 & port2, port1 & port3, port1 & port4, port2 & port3, port2 & port4 and port3 & port 4).


The Correlation-Coefficient_38.0/60.0GHz_ shown in Fig 7(a) and 7(b) signifies or describes the isolation between the communication channel, or also the correlation between the radiation patterns generated by the individual element of the MIMO_38.0/60.0GHz_ system [43]. The square of the correlation-coefficient (CC) yields ECC_38.0/60.0GHz_ and is calculated by the formation of radiation-patterns for two-element and is given by Equation 10

$$\begin{array}{c} \rho_{e}=\frac{{\left|\iint_{\text{4}\pi }\left[\vec{F_{Ant.\;\text{1}}}\;(\theta ,\phi )*\vec{F_{Ant.\;\text{2}}}\;(\theta ,\phi )\right]d\Omega \right|}^{\text{2}}}{\iint_{\text{4}\pi }{\left|F_{Ant.\;\text{1}}(\theta ,\phi )\right|}^{\text{2}}d\Omega \;\iint_{\text{4}\pi }{\left|F_{Ant.\;\text{2}}(\theta ,\phi )\right|}^{\text{2}}d\Omega } \end{array}$$ (10)

Where $\vec{F_{Ant.\;\text{1}}}\;(\theta ,\phi )$ and $\vec{F_{Ant.\;\text{2}}}\;(\theta ,\phi )$ shows the function of 3-D radiation pattern from the pool of multiple antenna elements within the MIMO_38.0/60.0GHz_ system (Ant. 1, Ant. 2….. Ant. i…..Ant. n). However, in real scenario, the ECC_38.0/60.0GHz_ is evaluated on extracted S-parameters (Ant. 1 & Ant. 2: S_11_, S_12_, S_21_, S_22_) and is calculated by the Equation 11 given below


$$\begin{array}{c} {\left|{CC}_{mmWave}\right|}^{\text{2}}={ECC}_{mmWave}={\left|\frac{\left|\overset{*}{\underset{aa}{S}}\;S_{ab}+\;\overset{*}{\underset{ba}{S}}\;S_{bb}\;\right|}{{\left|\left(\text{1}-{\left|S_{aa}\right|}^{\text{2}}-{\left|S_{ba}\right|}^{\text{2}}\right)\left(\text{1}-{\left|S_{bb}\right|}^{\text{2}}-{\left|S_{ab}\right|}^{\text{2}}\right)\right|}^{\text{1}/\text{2}}}\right|}^{\text{2}} \end{array}$$ (11)

Where ECC_ab_ is the correlation-coefficient between the antenna elements Ant. a and Ant. b. The maximum allowable CC_38.0/60.0GHz_ is 0.30, and the maximum ECC_mmWave_ is 0.50.

The measured mutual-coupling between the inter-spaced elements is below −20.0 dB, and also impedance matching better than −15.0 dB, the lower values of ECC are achieved between the range of 10 ⁻ ⁴–10 ⁻ ³. Also, the inter-space distance of 4.0 mm approximates to 0.8λg–1.2λg at 38–60 GHz, which indicates that the antenna appears physically compact, and it is electrically well separated at mmWave frequencies. Furthermore, the 180° orthogonal placement of the radiating elements reduces near-field overlap and promotes pattern diversity, while the full common ground suppresses surface-wave coupling. These factors collectively justify the low correlation observed in measurements. Finally, the uncertainty in measured results is also discussed, which has a much lesser impact in variation of ECC_mmWave_.

The DG_38.0/60.0GHz_ is another parameter to understand the performance of the MIMO_38.0/60.0GHz_ system. The diversity means in the MIMO_38.0/60.0GHz_ channel environment that the antenna elements receive the signals from different directions and with uncorrelated signals, a higher signal-to-noise ratio is expected, which indicates better reception of the signals. The Diversity Gain_38.0/60.0GHz_ shown in Fig 7(c) and 7(d) can be defined as the difference between the average-time SNR of the combined signals. The DG_38.0/60.0GHz_ is correlated to ECC_mmWave_ from Equation 12 given below.


$$\begin{array}{c} {DG}_{\text{38}.\text{0}/\text{60}.\text{0GHz }}=\sqrt{\text{1}-{\left|{ECC}_{mmWave}\right|}^{\text{2}}} \end{array}$$ (12)

The bandwidth offered by the proposed MIMO_38.0/60.0GHz_ antenna and the corresponding efficiency are not sufficient to understand the working of the MIMO configuration under diversity schemes. The TARC_38.0/60.0GHz_ shown in Fig 7(e) and 7(f) can be defined in simplified form as square-root of the total-reflected power (b_i_) to the square-root corresponding to the incident power (a_i_). Mathematically, it is given by Equation 13


$$\begin{array}{c} \overset{t}{\underset{a}{TARC}}=\frac{\sqrt{\sum_{i=\text{1}}^{N}{\left|b_{i}\right|}^{\text{2}}}}{\sqrt{\sum_{i=\text{1}}^{N}{\left|a_{i}\right|}^{\text{2}}}} \end{array}$$ (13)

Where ai and bi correspond to incident as well as reflected power-signals.

The TARC_38.0/60.0GHz_ calculation takes into account the coupling and random-signal combinations, and then the numeric value of TARC_38.0/60.0GHz_ is in the range between 0 and 1. For any two-port network (say port-q and port-b), TARC_38.0/60.0GHz_ (dB) is calculated from Equation 14


$$\begin{array}{c} {TARC}_{a,b}=\frac{\sqrt{(\left({\left|S_{aa}+S_{ab}e^{j\theta }\right|}^{\text{2}}\right)+\left({\left|S_{ba}+S_{bb}e^{j\theta }\right|}^{\text{2}}\right)}}{\sqrt{\text{2}}} \end{array}$$ (14)

The purpose of the integration of a greater number of radiating elements that are identical on the single-substrate is to increase the capacity of the channel. However, during transmission through the channel, the loss of information is expected but should be limited and is expressed by Channel-Capacity-Loss_38.0/60.0GHz_. The correlation between the transmitting antenna components exists in the MIMO_38.0/60.0GHz_ system, which can be reduced but cannot be ideally made zero. The higher value of SNR is the need for larger bandwidth, as shown by Equation 5 (Shannon-Capacity). Thus, the capacity loss is obtained by simulated and S-parameter with higher SNR in the uniform-multipath environment. The CCL_38.0/60.0GHz_ shown in Fig 7(g) and 7(h) is calculated by following Equation 15, Equation 16, and Equation 17


$$\begin{array}{c} {CCL}_{\text{38}.\text{0}/\text{60}.\text{0GHz }}=-{log}_{\text{2}}det\left(\alpha^{s}\right) \end{array}$$ (15)

$$\begin{array}{c} \rho_{mm}=\text{1}-\sum \overset{\text{4}}{\underset{n=\text{1}}{\mathrm{\backslash nolimits}}}{\left|S_{mn}\right|}^{\text{2}} \end{array}$$ (16)

$$\begin{array}{c} \rho_{ms}=-\left(\overset{*}{\underset{mm}{S}}S_{ms}+\overset{*}{\underset{sm}{S}}S_{ms}\right) \end{array}$$ (17)

Mean Effective Gain_38.0/60.0GHz_ (MEG_38.0/60.0GHz_ is calculated with the consideration of wireless-environment and under ideal conditions, the probabilistic model MEG_38.0/60.0GHz_ is calculated from Equation 18


$$\begin{array}{c} {MEG}_{\text{38}.\text{0}/\text{60}.\text{0GHz }}=\text{0}.\text{5}\times \left[\text{1}-\sum \overset{M}{\underset{j=\text{1}}{\mathrm{\backslash nolimits}}}{\left|S_{ij}\right|}^{\text{2}}\right] \end{array}$$ (18)

where M indicates the number of radiators printed on the top surface of the substrate (M = 4), I indicates the port-number, and the MEGport-1 is given as

MEG_38.0/60.0GHz_ shown in Fig 7(i), 7(j), 7(k), 7(l), 7(m), 7(n), 7(o), 7(p), 7(q), 7(r), 7(s) and 7(t) can be termed as the ratio of power received by MIMO_m38.0/60.0GHz_ antenna to that of the power received by ideal isotropic antenna. The improved diversity is calculated by taking the ratio of MEG_38.0/60.0GHz(port-1)_ to MEG_38.0/60.0GHz(port-2)_ which should be in the range between −3.0≤MEG_38.0/60.0GHz_ (dB)<-12.0.

Table 4 shows the averaged values of the diversity parameters ECC_38.0/60.0GHz_, DG_38.0/60.0GHz_, TARC_38.0/60.0GHz_, CCL_38.0/60.0GHz,_ and MEG_38.0/60.0GHz_. The table consists of simulated and measured averaged values at 38.0GHz and 60.0GHz which are compared with standard values, and the diversity-parameters are below the permissible standard values, indicating the proposed MIMO_38.0/60.0GHz_ antenna is suitable for faster-communication in the fading environment.

**Table 4:** The extracted simulated/measured diversity-parameters.

Average Values	Simulated		Measured		Standard Values
	n260 at 38.0GHz	n263 at 60.0GHz	n260	n263	
**ECC**_**38.0/60.0GHz**_	2.32 × 10^−4^	3.24 × 10^−4^	2.78 × 10^−4^	2.93 × 10^−4^	<0.50
**DG**_**38.0/60.0GHz**_ **(dB)**	≈10.0	≈10.0	≈10.0	≈10.0	10.0
**TARC**_**38.0/60.0GHz**_ **(dB)**	−9.03	−7.154	−7.358	−9.12	0
**CCL**_**38.0/60.0GHz**_ **(b/s/Hz)**	0.829 × 10^−2^	1.97 × 10^−2^	1.58 × 10^−2^	1.83 × 10^−2^	0.40
**MEG**_**_port1**_ **(dB)**	≈-3.0	≈-3.0	≈-3.0	≈-3.0	−3.0≤MEG_38.0/60.0GHz_(dB)<-12.0.

## 5. 4-Port mmWave MIMO_38.0/60.0GHz_ configuration with conformal, SAR, and Far-Field results

**Fig 8:**
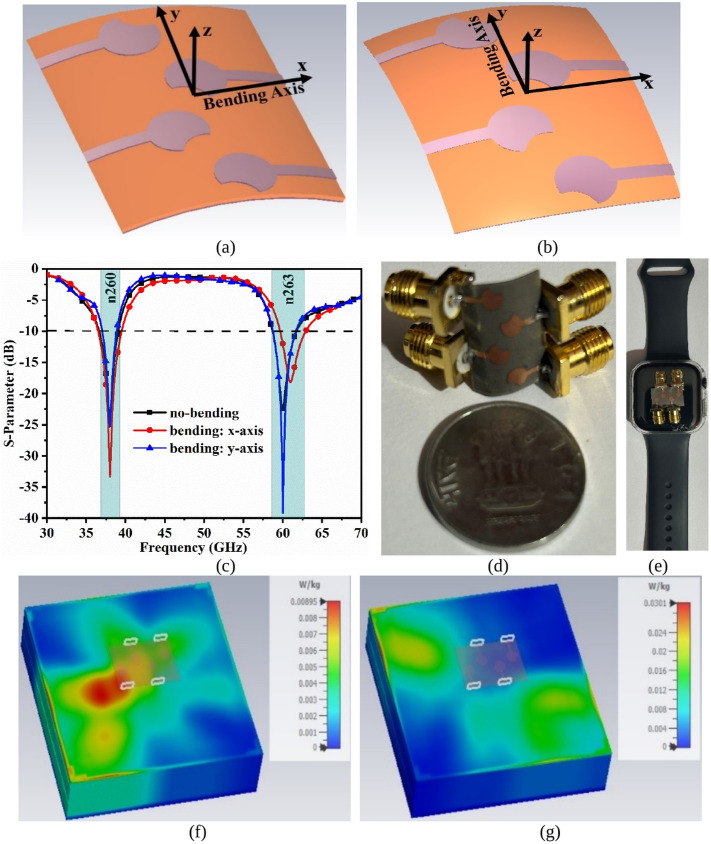
Bending and SAR analysis of Four-port MIMO antenna. **(a)** Bending of the antenna at 45° in x-axis **(b)** Bending of the antenna at 45° in y-axis **(c)** S-parameter of bending analysis **(d)** Physical bending of the antenna **(e)** MIMO antenna on Smart Watch; SAR analysis at (f) 38.0GHz (g) 60.0GHz.

Fig 8 shows the bending analysis and SAR_38.0/60.0GHz_ calculation of the proposed MIMO_38.0/60.0GHz_ antenna for on-off body applications. Fig 8(a) and Fig 8(b) show the bending of the antenna in both x and y-axis direction at an angle of 45°. For MIMO_38.0/60.0GHz_ with no bending offers impedance bandwidth of 36.65GHz-39.06GHz with maximum resonance at 37.99GHz for n260 band and 58.71GHz-61.76GHz with resonance corresponding at 59.99GHz in partial-n263 band. The bending in x-axis direction shown in Fig 8(c) shows the capability of the antenna maintaining the bandwidth for both the narrow-band corresponding to 36.52GHz-39.49GHz and 59.67GHz-62.92GHz.

Fig 8(c) also records the bandwidth of 37.05GHz-38.87GHz (n260) and 58.75GHz-61.66GHz (n263) is maintained when the antenna is bent at 45° in the y-axis direction. Fig 8(d) shows the physical-bending of the antenna, and Fig 8(e) signifies that the ultra-compact size of the antenna can be easily integrated with a wearable smart-watch for future 5G applications. The MIMO antenna, as discussed, is designed for on-off body applications and hence becomes essential to perform the Specific-Absorption-Rate_38.0/60.0GHz_ on the 38.0GHz and 60.0GHz mmWave band, which is also shown in Fig 8(f) and 8(g). The SAR_m38.0/60.0GHz_ values at 38.0GHz corresponds to 0.00895 W/kg and 0.0301 W/kg at 60.0 GHz. These two SAR_38.0/60.0GHz_ values are well below the 1.60 W/kg standard value.

The far-field measurements were recorded within the anechoic chamber, where standard gain horn antennas were used as the transmitter, satisfying the Fraunhofer distance between the transmitter (Horn antenna) and receiver (proposed MIMO antenna). The proposed MIMO antenna was mounted on low-permittivity (to avoid measurement effect) foam support with the rotary positioner rotated in E-plane and H-plane for 2D radiation patterns. However, in spite of all the care taken for measured results, the measurement uncertainty occurs related to VNA amplitude uncertainty at mmWave of ±0.50 dB, phase uncertainty of ±3∘. Also, the fabrication tolerances do affect the desired results with sub-millimeter dimensions of ±0.02 mm, which observes the shift in the resonance either at 38.0 GHz, 60.0 GHz, or both.

**Fig 9:**
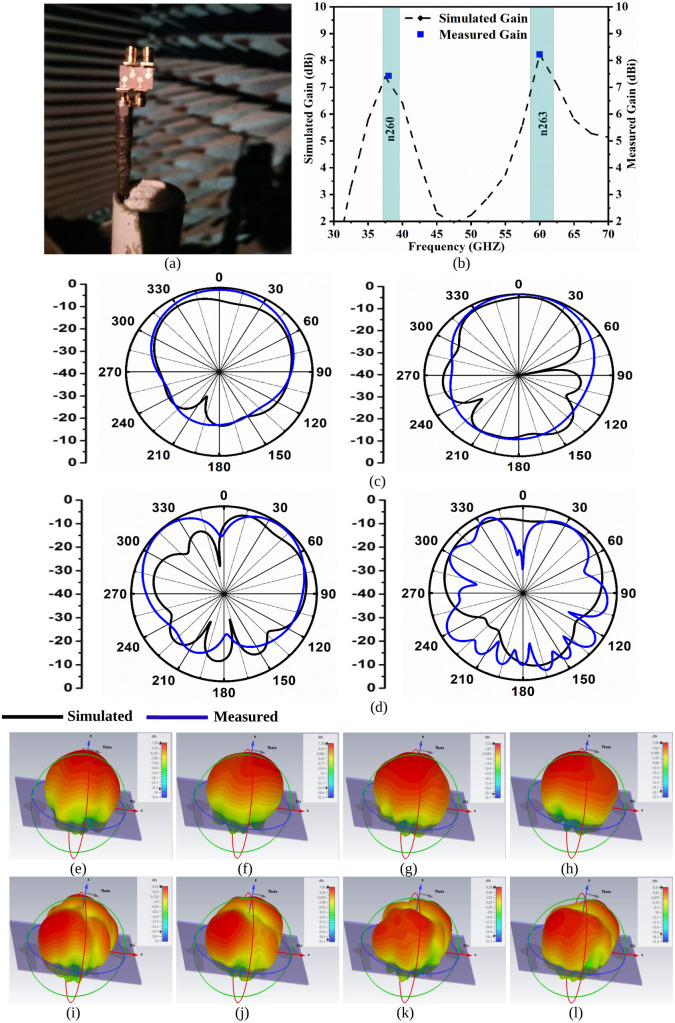
Far-Field Analysis. **(a)** Prototype placed within Anechoic Chamber **(b)** Simulated and Measured Gain (dBi); 2-D Radiation Pattern at (c) 38.0GHz (d) 60.0GHz; 3D-radiation pattern at 38.0 GHz for **(e)** Port-1 **(f)** Port-2 **(g)** Port-3 **(h)** Port-4; 3D-radiation pattern at 60.0 GHz for **(i)** Port-1 **(j)** Port-2 **(k)** Port-3 **(l)** Port-4.

The far-field analysis of the proposed MIMO_38.0/60.0GHz_ antenna is plotted in Fig 9. Fig 9(a) shows the placement of the prototype for gain as well as for 2-D radiation measurement. The average simulated gain for the n260 and partial-n263 band corresponds to 6.532dBi & 6.978dBi. The measured gain at 38.0GHz is 7.426dBi and 60.0GHz corresponds to 8.222dBi. Fig 9(c) and 9(d) show the simulated and measured 2-D radiation patterns, which are plotted at 38.0Ghz and 60.0GHz. The elevation plane corresponds to ϕ = 0° which is the E-plane, and ϕ = 90° corresponds to the H-plane. The designed MIMO_38.0/60.0GHz_ antenna needs to inherit high gain as the application bands target 5G applications. The radiation-patterns in both planes correspond to the spreading of the patterns, which suggests that the proposed antenna is feasible in applications requiring a larger area. The simulated and measured results offer an aligned pattern, but minor inconsistencies do occur due to reasons such as losses in cable, errors in the manufacturing of the prototype, and losses related to measurements.

Fig 9 also includes the 3D radiation patterns for 38.0 GHz & 60.0 GHz obtained for Port-1, Port-2, Port-3 & Port-4. Fig 9(e), 9(f), 9(g) & 9(h) shows the 3D radiation for Port-1 to Port-4 with maximum peak-realized-gain variation of 7.39 dBi to 7.78 dBi as tabulated in Table 5. Also, Fig 9(i), 9(j), 9(k) & 9(l) shows the 3D radiation for Port-1 to Port-4 with maximum peak-realized-gain variation of 7.93 dBi to 8.28 dBi as tabulated in Table 5.

**Table 5:** Comparison of dual-band mmWave MIMO antennas (2020–2024).

Frequency	Port-1	Port-2	Port-3	Port-4
38.0 GHz	7.39 dBi	7.78 dBi	7.31 dBi	7.66 dBi
60.0 GHz	8.01 dBi	7.93 dBi	8.28 dBi	8.14 dBi

Table 6 shows the comparison of the proposed dual-band MIMO_38.0/60.0GHz_ antenna with similar published work. The size of the antenna is ultra-compact in its four-port configuration. Also, there is very little literature available with dual-band dedicated to 38.0GHz and 60.0GHz mmWave, which was motivational for this work. Also, Reference [3] is subjected to SAR analysis, but the size of the antenna is large (3600 mm^2^) compared with the proposed work (192 mm^2^). The proposed work outperforms the other reported work in comparison with size and is proposed for future 5G and ISM bands.

**Table 6:** Comparison of the proposed work with previously published work.

Ref. No.	Size (mm^2^)/ Year/ Substrate Type	Ports/ Bandwidth/ Common Ground (Yes/No)	Isolation (dB)	ECC	DG (dB)	TARC (dB)	CCL b/s/Hz	Gain	Conformal Capability	SAR Analysis/Values (W/Kg) w.r.t. Frequency (GHz)	Potential Applications
[1]	25.95 × 25.95 (4.85λ_0_×4.85 λ_0_) 2023 Rigid	4 37.20-39.20 NO	<−25.0	<0.005	>9.99	NC	<0.40	10.0	NO	NO	5G mmWave
[2]	20.0 × 40.0 (3.14λ_0_×6.29λ_0_) 2022 Rigid	2 37.29-38.64	<−40.0	<1 × 10^−4^	>9.9999	NC	<0.20	12.80	NC	NC	5G mmWave
[3]	60.0 × 60.0 (6.92λ_0_×6.92λ_0_) 2023 Rigid	4 27.35-30.40 36.98-39.398	<−25.0	<0.0035	>9.982	NC	<0.40	8.14	NC	0.702 at 28.0GHz 0.623 t 38.0GHz	5G mmWave
[4]	30.0 × 30.0 (3.64λ_0_×3.64λ_0_) 2022 Rigid	4 25.91-30.22 35.46-40.45	<−17.0	<0.0041	≈10.0	NC	<0.35	6.32	NA	NA	5G mmWave
[5]	14.76 × 8.38 (1.02λ_0_×1.80λ_0_) 2023 Flexible	2 27.0-51.0	<−20.0	<2.5 × 10^−5^	≈10.0	<−30.0	<0.30	6.00	NC	0.963 at 28.0GHz 0.583 at 38.0GHz	5G mmWave
[9]	6.00 × 8.00 (0.68λ_0_×0.90λ_0_) 2022 Flexible	1 26.75-30.31 35.83-41.22	NA	NA	NA	NA	NA	4.50	NC	NC	5G mmWave
[10]	22.5 × 24.0 (2.62λ_0_×2.79λ_0_) 2021 Rigid	4 27.6-28.6 37.4-38.6	<−27.5	<0.0005	NC	NC	NC	7.90	NO	NO	5G mmWave
[18]	17.5 × 22.0 (4.17λ_0_×5.24λ_0_) 2020 Rigid	1 56.5-65.2	NA	NA	NA	NA	NA	16.0	NO	NO	ISM
[44]	6.0 × 6.0 (0.51λ_0_×0.51λ_0_) 2024 Flexible	1 25.0-26.5 37.0-39.5	NA	NA	NA	NA	NA	7.40	NO	0.063 at 25.5GHz 0.0206 at 38.0GHz	5G mmWave
[45]	20.0 × 20.0 (1.87λ_0_×1.87λ_0_) Rigid	4 27.53-28.16 30.13-30.81	<−18.0	<0.002	>9.98	NC	<0.40	5.90	NO	NO	5G mmWave
[46]	30.0 × 30.0 (3.21λ_0_×3.21λ_0_) Rigid	4 26.95-36.77	<−18.0	<0.0028	>9.96	<−10.0	NC	5.11	NO	NO	5G mmWave
[47]	10.8 × 9.0 (1.22λ_0_×1.02λ_0_) Rigid	4 24.0-50.0	<−30.0	<6 × 10^−3^	>9.99	<−25.0	NC	10.35	NO	NO	5G mmWave
[48]	20.0 × 24.0 (0.61λ0×0.74λ0) Flexible	4 7.27-34.32 46.54-71.52	>15.0	<0.01	≈10.0	<−4.0	<0.12	5.12	YES	1.01 at 10.0 GHz 0.28 at 15.0 GHz 0.475 at 15.0 GHz 0.68 at 26.0 GHz 0.588 at 28.0 GHz 0.301 at 60.0 GHz	5G mmWave
*P	12.0 × 16.0 (1.85λ_0_×2.47λ_0_) Flexible	4 36.6-39.12 58.48-61.48	>20.0	<2.78 × 10−4 <2.93 × 10−4	≈10.0 ≈10.0	<−7.358 <−9.12	<1.58 × 10−2 <1.83 × 10−2	6.532 6.978	YES	0.00895 at 38.0GHz 0.0301 at 60.0GHz	5G mmWave ISM

*P-Proposed work.

## 6. Conclusions and future scope of work

A four-port dual-band MIMO38.0/60.0GHz antenna is investigated for on-off body applications. In addition to the simulation results, a prototype is also fabricated to validate and compare the measured and simulated results. The measured bandwidth of 2.52 GHz (n260) and 30.0 GHz (n263) is achieved by using ultra-compact size MIMO_38.0/60.0GHz_ antenna fabricated on thin substrate with overall area of the antenna to be 192 mm^2^. The four-radiating patches are placed adjacent to each other and 180° orientation with circular-etched patches and ground, maintaining isolation of more than 20.0dB between neighboring radiating elements. Furthermore, the measured gain corresponds to 6.532dBi/38.0GHz and 6.978dBi/60.0GHz with diversity performance including ECC_38.0/60.0GHz_ < 0.000278, DG_38.0/60.0GHz_≈10.0dB, TARC_38.0/60.0GHz_ < −7.538dB, CCL_38.0/60.0GHz_ < 0.0158 b/s/Hz and MEG_38.0/60.0GHz_≈-3.0 dB. The flexible capability of the MIMO_38.0/60.0GHz_ antenna is also studied with bending at 45° in the x-axis and y-axis direction, and minor deviation of S-parameter is observed in both the bands. The SAR_38.0/60.0GHz_ value of 0.00895 W/Kg at 38.0GHz and 0.0301 W/Kg at 60.0GHz confirms the integration of the MIMO_38.0/60.0GHz_ antenna for on-off body application for future 5G. The prospect of the work can be extended to the addition of other FR2 bands, and also the work can be extended with additional technology inclusion, such as meta-material, electromagnetic band-gap structure, and deployment of a frequency-selective surface to reduce the SAR.

The proposed MIMO antenna is designed primarily for compact 5G FR2 module level integration such as mmWave front-end modules or small-form-factor user equipment operating at 38 GHz and 60 GHz.

## Supporting information

S1 File SF.
(DOCX)
