# Supplementary material for: Experimental investigation of four-port MIMO-Dual-Band (MDB) antenna for n260/n263 mmWave bands with analysis including conformal & specific-absorption-rate
Source: PLoS One. 2026 May 29;21(5):e0350033. doi: 10.1371/journal.pone.0350033 (PMC13221041; doi:10.1371/journal.pone.0350033)
Supplement: S1 File — SF. (DOCX) [file pone.0350033.s001.docx]

**Table 1. Optimal Dimensions of the Proposed Antenna.**

| Parameter | Description | Value (mm) |
| --- | --- | --- |
| **W~sub~** | Width of Substrate | 6.00 |
| **L~sub~** | Length of Substrate | 8.00 |
| **W~m~** | Width of Microstrip Feed Line | 0.80 |
| **L~m~** | Length of Microstrip Feed Line | 3.50 |
| **R~D~** | Radius of Driven Patch | 1.80 |
| **R** | Radius of Inner Etched Circular Slot | 1.36 |
| **L~g~** | Length of Ground Plane | 8.00 |
| **W~g~** | Width of Ground Plane | 6.00 |

**Table 2. Dimensional Effects on Frequency Control.**

| Related Parameter | Effect | Frequency Control |
| --- | --- | --- |
| **R~D~** (Driven Patch Radius) | Controls the fundamental TM~11~ mode. | Corresponds to 38.0 GHz |
| **R** (Inner Etched Radius) | Perturbs a higher-order mode, creating the second resonance. | Corresponds to 60.0 GHz |
| Ground Dimensions (L~g~, W~g~) | Affects bandwidth and impedance matching for both bands. | Dual-band operation |
| Substrate Height (t) | Affects bandwidth and Q-factor for both bands. | Dual-band operation |

**Table 3. Electrical Properties of Human Tissues at 38.0 GHz and 60.0 GHz.**

| Tissue | Frequency | Permittivity (ε~r~) | Conductivity (S/m) | Loss Tangent (tan δ) |
| --- | --- | --- | --- | --- |
| **Skin** | 38.0 GHz | 123 | 31.0 | 1.7941 |
|  | 60.0 GHz | 7.98 | 36.4 | 1.3673 |
| **Fat** | 38.0 GHz | 5.33 | 6.36 | 0.29331 |
|  | 60.0 GHz | 4.40 | 8.39 | 0.26925 |
| **Muscle** | 38.0 GHz | 19.1 | 41.8 | 1.0382 |
|  | 60.0 GHz | 12.9 | 52.8 | 1.231 |

**Table 4. Summary of Simulated and Measured MIMO Diversity Parameters.**

| Parameter | Simulated | Measured | Standard Value |  |  |
| --- | --- | --- | --- | --- | --- |
|  | **38.0 GHz** | **60.0 GHz** | **38.0 GHz** | **60.0 GHz** |  |
| **ECC** | 2.32×10^-4^ | 3.24×10^-4^ | 2.78×10^-4^ | 2.93×10^-4^ | < 0.50 |
| **DG (dB)** | ≈10.0 | ≈10.0 | ≈10.0 | ≈10.0 | ≈10.0 |
| **TARC (dB)** | -9.03 | -7.154 | -7.358 | -9.12 | 0 (Ideal) |
| **CCL (b/s/Hz)** | 0.829×10^-2^ | 1.97×10^-2^ | 1.58×10^-2^ | 1.83×10^-2^ | < 0.40 |
| **MEG~port1~ (dB)** | ≈-3.0 | ≈-3.0 | ≈-3.0 | ≈-3.0 | -3.0 to -12.0 |

**Table 5. Simulated Peak Realized Gain (dBi) per Port.**

| Frequency | Port-1 | Port-2 | Port-3 | Port-4 |
| --- | --- | --- | --- | --- |
| **38.0 GHz** | 7.39 dBi | 7.78 dBi | 7.31 dBi | 7.66 dBi |
| **60.0 GHz** | 8.01 dBi | 7.93 dBi | 8.28 dBi | 8.14 dBi |

**Table 6. Comparison of Design Specifications with Previously Published Works.**

| Ref. | Size (mm²)/ λ₀² | Year / Substrate Type | Ports / Bands (GHz) / Common Ground |
| --- | --- | --- | --- |
| [1] | 25.95×25.95 (4.85λ₀×4.85λ₀) | 2023 / Rigid | 4 / 37.2-39.2 / No |
| [2] | 20.0×40.0 (3.14λ₀×6.29λ₀) | 2022 / Rigid | 2 / 37.29-38.64 / -- |
| [3] | 60.0×60.0 (6.92λ₀×6.92λ₀) | 2023 / Rigid | 4 / 27.35-30.40, 36.98-39.398 / -- |
| [4] | 30.0×30.0 (3.64λ₀×3.64λ₀) | 2022 / Rigid | 4 / 25.91-30.22, 35.46-40.45 / -- |
| [5] | 14.76×8.38 (1.02λ₀×1.80λ₀) | 2023 / Flexible | 2 / 27.0-51.0 / -- |
| [9] | 6.00×8.00 (0.68λ₀×0.90λ₀) | 2022 / Flexible | 1 / 26.75-30.31, 35.83-41.22 / NA |
| [10] | 22.5×24.0 (2.62λ₀×2.79λ₀) | 2021 / Rigid | 4 / 27.6-28.6, 37.4-38.6 / -- |
| [18] | 17.5×22.0 (4.17λ₀×5.24λ₀) | 2020 / Rigid | 1 / 56.5-65.2 / NA |
| [37] | 6.0×6.0 (0.51λ₀×0.51λ₀) | 2024 / Flexible | 1 / 25.0-26.5, 37.0-39.5 / NA |
| [43] | 20.0×20.0 (1.87λ₀×1.87λ₀) | -- / Rigid | 4 / 27.53-28.16, 30.13-30.81 / -- |
| [44] | 30.0×30.0 (3.21λ₀×3.21λ₀) | -- / Rigid | 4 / 26.95-36.77 / -- |
| [45] | 10.8×9.0 (1.22λ₀×1.02λ₀) | -- / Rigid | 4 / 24.0-50.0 / -- |
| [46] | 20.0×24.0 (0.61λ₀×0.74λ₀) | -- / Flexible | 4 / 7.27-34.32, 46.54-71.52 / -- |
| ***P** * | **12.0×16.0** | **2024 / Flexible** | **4 / 36.6-39.12, 58.48-61.48 / YES** |

**Table 7. Comparison of MIMO Performance Metrics with Previously Published Works.**

| Ref. | Iso. (dB) | ECC | DG (dB) | TARC (dB) | CCL (b/s/Hz) | Gain (dBi) |
| --- | --- | --- | --- | --- | --- | --- |
| [1] | <-25.0 | <0.005 | >9.99 | NC | <0.40 | 10.0 |
| [2] | <-40.0 | <1×10⁻⁴ | >9.9999 | NC | <0.20 | 12.80 |
| [3] | <-25.0 | <0.0035 | >9.982 | NC | <0.40 | 8.14 |
| [4] | <-17.0 | <0.0041 | ≈10.0 | NC | <0.35 | 6.32 |
| [5] | <-20.0 | <2.5×10⁻⁵ | ≈10.0 | <-30.0 | <0.30 | 6.00 |
| [9] | NA | NA | NA | NA | NA | 4.50 |
| [10] | <-27.5 | <0.0005 | NC | NC | NC | 7.90 |
| [18] | NA | NA | NA | NA | NA | 16.0 |
| [37] | NA | NA | NA | NA | NA | 7.40 |
| [43] | <-18.0 | <0.002 | >9.98 | NC | <0.40 | 5.90 |
| [44] | <-18.0 | <0.0028 | >9.96 | <-10.0 | NC | 5.11 |
| [45] | <-30.0 | <6×10⁻³ | >9.99 | <-25.0 | NC | 10.35 |
| [46] | >15.0 | <0.01 | ≈10.0 | <-4.0 | <0.12 | 5.12 |
| ***P** * | **>20.0** | **<2.78×10⁻⁴ (38) <2.93×10⁻⁴ (60)** | **≈10.0** | **<-7.358 (38) <-9.12 (60)** | **<1.58×10⁻² (38) <1.83×10⁻² (60)** | **6.53 (38) 6.98 (60)** |

**Table 8. Comparison of Advanced Features (Conformal Capability and SAR) with Previously Published Works.**

| Ref. | Conformal Capability | SAR (W/Kg) / Freq. (GHz) |
| --- | --- | --- |
| [1] | NO | NO |
| [2] | NC | NC |
| [3] | NC | 0.702 (28.0) / 0.623 (38.0) |
| [4] | NA | NA |
| [5] | NC | 0.963 (28.0) / 0.583 (38.0) |
| [9] | NC | NC |
| [10] | NO | NO |
| [18] | NO | NO |
| [37] | NO | 0.063 (25.5) / 0.0206 (38.0) |
| [43] | NO | NO |
| [44] | NO | NO |
| [45] | NO | NO |
| [46] | YES | 1.01 (10.0), 0.28 (15.0), 0.475 (15.0), 0.68 (26.0), 0.588 (28.0), 0.301 (60.0) |
| ***P** * | **YES** | **0.00895 (38.0) / 0.0301 (60.0)** |

*NC: Not Considered / Not Clearly Stated, NA: Not Applicable, λ₀: Free-space wavelength at the lowest operating frequency. P-Proposed work.
